# Improved Active Harmonic Current Elimination Based on Voltage Detection

**DOI:** 10.1371/journal.pone.0157057

**Published:** 2016-06-13

**Authors:** Tianyuan Tan, Shuan Dong, Yingwei Huang, Jian Liu, Jian Le, Kaipei Liu

**Affiliations:** 1 School of Electrical Engineering, Wuhan University, Wuhan, Hubei, China; 2 Department of Electrical and Computer Engineering, University of British Columbia, Vancouver, BC, Canada; Chongqing University, CHINA

## Abstract

With the increasing penetration of power electronic equipment in modern residential distribution systems, harmonics mitigation through the distributed generation (DG) interfacing converters has received significant attention. Among recently proposed methods, the so-called active resonance damper (ARD) and harmonic voltage compensator (HVC) based on voltage detection can effectively reduce the harmonic distortions in selected areas of distribution systems. However, it is found out that when traditional ARD algorithm is used to eliminate harmonic current injected by non-linear loads, its performance is constrained by stability problems and can at most eliminate half of the load harmonic currents. Thus, inspired by the duality between ARD and HVC, this paper presents a novel improved resistive active power filter (R-APF) algorithm based on integral-decoupling control. The design guideline for its parameters is then investigated through carefully analyzing the closed-loop poles’ trajectory. Computer studies demonstrate that the proposed algorithm can effectively mitigate the load harmonic currents and its performance is much better than traditional ARD based on proportional control.

## Introduction

With the rapid development of power electronics technology, energy efficient but harmonic producing power converters have been widely used in both commercial and residential installations, including motor-type loads fed through variable frequency drives (VFDs), personal computers (PCs), television sets (TVs), fluorescent lamps(FLs), etc. Despite the relative small capacity of each power electronic device, these non-linear loads combined are known to contribute considerable harmonics into the power systems, resulting in extra losses, adverse impact on system stability as well as other equipment [1–3].

At the same time, increasing number of distributed generation (DG) units such as photovoltaic panels, wind turbines, and energy storage devices are being connected to the grid [4], [5]. Most of these DG systems are connected to the grid through power electronic converters [6]. If designed and controlled improperly, the DG interfacing converters may also introduce harmonics into the power system and cause power quality concerns [7]. On the contrary, if designed and controlled properly, these DG interfacing converters can potentially provide many ancillary services to improve the system efficiency and power quality, including the power factor compensation and system harmonic compensation, etc. [8]

Although shunt active power filters (APF) have been researched and put into practice for decades, most of the research are focused on their use for a specific harmonic-producing customer. Typically, traditional shunt APF is controlled to inject harmonic currents with the same characteristics (amplitude and frequency) and opposite polarity of the harmonic currents drawn by the distorting load. The APFs must be installed in the vicinity of the non-linear loads. However, the locating place of DG interfacing converters are mainly decided by other considerations and are most often than not far away from the non-linear loads or the entrance of the feeder. So, traditional harmonic mitigating strategy based on current detection does not suit these converters anymore. Alternatively, two novel control schemes based on harmonic voltage detection have been proposed, namely the active resonance damper (ARD) or resistance-active power filter (R-APF) [9] and the harmonic voltage compensator (HVC) [10][11]. Instead of injecting harmonic currents to compensate those drawn by distorting loads, the R-APF acts as a small harmonic resistance installed at the end of a radial line to damp harmonic resonance between power factor correction capacitors and system inductances. The HVC directly generates the harmonic voltages to compensate those at the point of common coupling. Both methods can effectively mitigate harmonics caused by harmonic voltage source on the system side, without the need to detect harmonic currents of the load (or the total harmonic currents injected to the utility after compensation).

This paper considers the distribution system of a representative educational building (which has considerable non-linear loads), and investigates into the active harmonic mitigation algorithm which is based on voltage detection and suitable for DG interfacing converters. It is found out that although traditional proportional R-APF algorithm can extend to eliminate harmonic current injected by non-linear loads, its performance is constrained by stability problems and can at most eliminate half of the load harmonic currents. Thus, inspired by the iterative HVC algorithm, this paper proposes a novel improved R-APF algorithm, which is based on integral-decoupling control in the synchronous rotating reference frame at each harmonic frequency. The design of *d-q* axes decoupling control and the selection of integral coefficient are then analyzed in details. Finally, computer simulations are presented to validate the proposed methodology.

## Configuration of the Subject System

**Fig 1:**
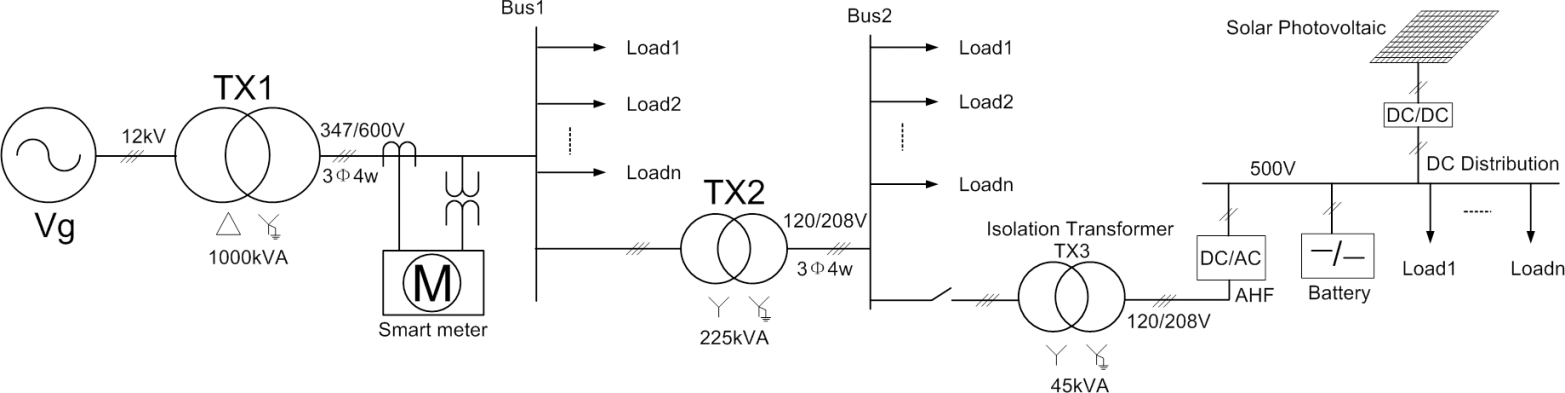
Configuration of the DG system in the representative educational Building.

For the purpose of this paper, the integrated ac-dc system of a representative educational Building is considered as depicted in Fig 1. Therein, the ac-dc system consists of two main parts, i.e., the ac distribution system connected to utility grid through step-down transformers, and the dc system comprising a large volume of battery storage system and a set of solar PV panels. In particular, the dc system can work as an islanding network, or connect to the ac system through the interfacing DC/AC converter. Additionally, a smart meter is installed at the outlet of the building’s main transformer TX1, which monitors and records the total active power and reactive power consumed by the building, as well as the harmonic voltage at Bus 1 and the total harmonic currents injected into the grid.

**Fig 2:**
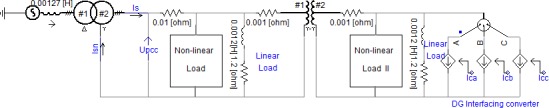
Simplified PSCAD simulation model of the DG system depicted in Fig 1.

The simplified equivalent circuit of this subject system is then modeled in PSCAD as shown in Fig 2. Since the background harmonics from the ac grid are typically small, the harmonic currents are assumed to be generated by non-linear loads within the building. Therein, they can be approximated as two lumped equivalent harmonic current sources connected to Bus 1 and 2, respectively. Similarly, the linear loads are lumped into two equivalent *R-L* loads as shown in Fig 2. In order to mitigate harmonic currents, the DG interfacing converters is controlled as a three-phase controlled current source at the each harmonic frequency. The methods to control the voltage source converter as a controlled current source have been well studied in the literature, including algorithms such as hysteresis control, ramp comparison control, and deadbeat control [12]. Therefore, this paper will only focus on how to get the reference value of harmonic current for the inner control loop.

## Harmonic Elimination Methods based on Voltage Detection

### A.The Duality between R-APF and HVC

Originally designed to damp harmonic propagation caused by harmonic voltage source on the system side [9], the R-APF has been lately used as harmonic compensation methods to eliminate harmonic currents from non-linear loads on the customer side [13]. In contrast, the HVC [10], [11] was proposed as a voltage-control-method based harmonic control method suitable for DG units which are desired to provide direct voltage and frequency support for loads in micro grids. In fact, HVC and R-APF are duals, harmonic voltage compensation instructions for HVC can be easily converted into harmonic current compensation commands for R-APF by simply converting the HVC’s Thevenin equivalent circuit into R-APF’s Norton equivalent circuit, and vice versa. Therefore, here we will only focus on the R-APF algorithm.

### B. Shortcomings of Traditional R-APF Algorithms

**Fig 3:**
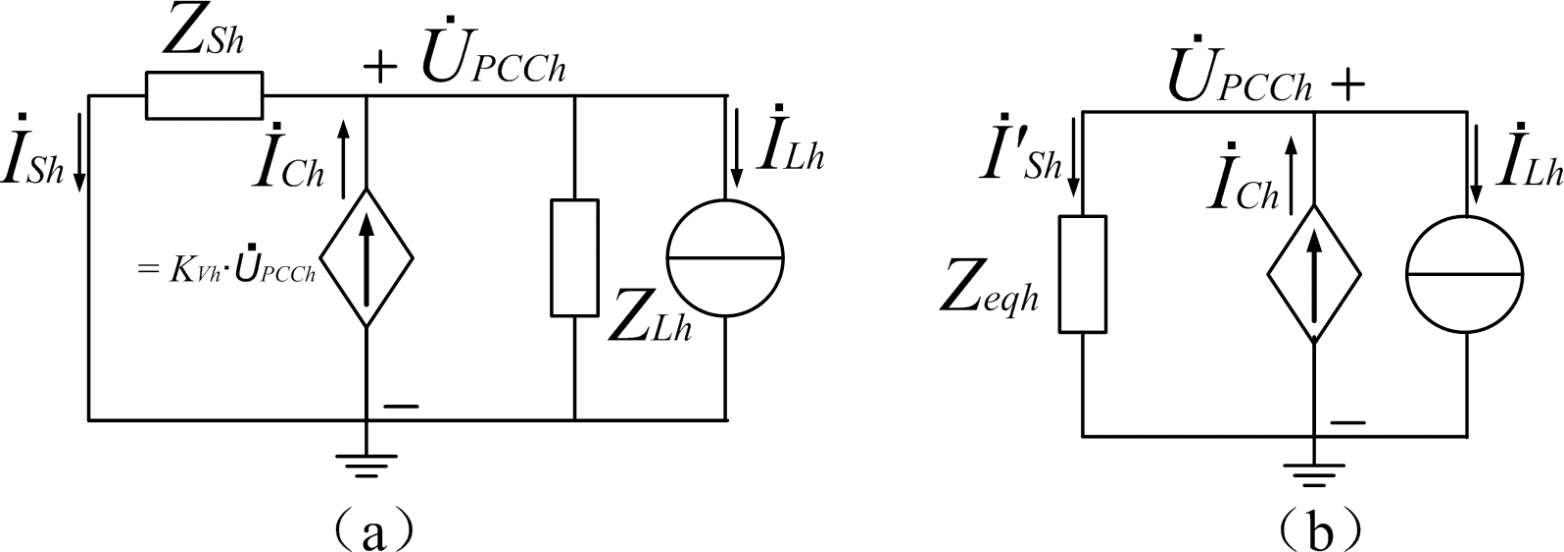
Principle circuit for R-APF: (a) per-phase equivalent circuit; (b) simplified.

Traditional R-APF algorithm [13] controls the converter’s output current and make it proportional to the PCC voltage at the harmonic frequencies, as shown in Fig 3. Wherein,

*Z*_*sh*_ is the harmonic impedance of the supply network at order *h*

${\dot{I}}_{Sh}$ is the harmonic current phasor inject into the supply network at order *h*

${\dot{I}}_{Lh}$ is the current phasor for the equivalent harmonic current source of the nonlinear loads at order *h*

*Z*_*Lh*_ is the equivalent linear load impedance at order *h*

${\dot{I}}_{Ch}$ is the injected compensation current phasor of R-APF at order *h* and its value is set to *K*_vh_* ${\dot{U}}_{PCCh}$, where *K*_vh_ is a fix real number

${\dot{U}}_{PCCh}$ is the harmonic voltage phasor of PCC at order *h*

*Z*_eqh_ is the parallel equivalent impedance of *Z*_sh_ and *Z*_Lh_

${\dot{I\prime }}_{Sh}$ is the current phasor injected to the equivalent parallel impedance, the relationship between ${\dot{I}}_{sh}$ and ${\dot{I^{\prime }}}_{sh}$ is ${\dot{I}}_{sh}={\dot{I^{\prime }}}_{sh}∙Z_{Lh}/\left(Z_{Lh}+Z_{Lh}\right)$

Applying KCL and KVL to the circuit in Fig 3(A) yields

$${\dot{I}}_{Sh}=-{\dot{I}}_{Lh}\cdot \frac{1}{1+Z_{Sh}/Z_{Lh}-{K_{Vh}\cdot Z}_{Sh}}.$$ (1)

As shown in (1), *I*_*sh*_ (the amplitude of *phasor* ${\dot{I}}_{sh}$) decreases with a larger *K*_Vh_ for any fixed *Z*_sh_ and *Z*_Lh_. If *K*_Vh_ tends to infinite, both *I*_*sh*_ and *U*_*PCCh*_ (the amplitude of *phasor* ${\dot{U}}_{PCCh}$) would tend to be zero. That’s how the R-APF eliminates the non-linear load currents.

However, in practical systems the digital controller is used and the corresponding discrete-time compensation algorithm yields

$$i_{Ch}(k)=K_{Vh}∙u_{PCCh}(k-1).$$ (2)

Where, *k* is the discrete sample and control interval, *i*_*Ch*_ *and u*_*PCCh*_ are the instantaneous value of phasor ${\dot{I}}_{Ch}$ and ${\dot{U}}_{PCCh}$ respectively.

It means the R-APF can only adjust its present output according to the last sample value instead of the present value, because it needs time to sample, calculate, and generate outputs. I.e., there exists control delay. Unfortunately, the control delay, no matter how small it can be, will always lead to stability problems.

Applying KCL and KVL to the circuit in Fig 3(A), the mathematical model of discrete system can be obtained as

$${\dot{U}}_{pcch}(k)={\dot{I}}_{Sh}(k)∙Z_{Sh},$$ (3)

$${\dot{I}}_{Sh}(k)=[{\dot{I}}_{Ch}(k)-{\dot{I}}_{Lh}(k)]\cdot Z_{Lh}/(Z_{Sh}+Z_{Lh}).$$ (4)

Substitute Eqs (2) and (3) into Eq (4) yields

$${\dot{I}}_{Sh}(k)={\dot{I}}_{Lh}(k)\cdot \frac{Z_{Lh}}{Z_{Sh}+Z_{Lh}}-{\dot{I}}_{sh}(k-1)\cdot K_{Vh}∙\frac{Z_{Sh}∙Z_{Lh}}{Z_{Sh}+Z_{Lh}}.$$ (5)

It’s obviously that the stable convergence condition for this discrete system described by Eq (5) is

$$|K_{Vh}∙Z_{eqh}|<1$$ (6)

As |Z_sh_|<<|Z_Lh_| holds most cases, and R-APF can be regarded as a resistor with the value of 1/*K*_Vh_, from Fig 3 we have

$$I_{Sh}=I_{Lh}\cdot \left|\frac{\frac{1}{Z_{Sh}}}{\frac{1}{Z_{Sh}}+K_{Vh}+\frac{1}{Z_{Lh}}}\right|>I_{Lh}\cdot \left|\frac{\frac{1}{Z_{Sh}}}{\frac{1}{Z_{Sh}}+\frac{1}{\left|Z_{eqh}\right|}+\frac{1}{Z_{Lh}}}\right|>0.5I_{Lh}.$$ (7)

Where, *I*_*sh*_ and *I*_*Lh*_ *are the RMS amplitude of phasor* ${\dot{I}}_{Sh}$ and ${\dot{I}}_{Lh}$ respectively.

It means that the optimal performance that traditional R-APF can achieve is eliminating about 50% of the load harmonic currents. Note that if *K*_Vh_ is too large, system will become unstable.

### C. A novel improved integral control R-APF algorithm

In order to improve R-APF algorithm’s performance, this paper proposes an integral control by its duality to the iterative HVC method [11].

$$i_{Ch}(k)=i_{Ch}(k-1)+K_{Vh}∙u_{PCCh}(k-1)$$ (8)

As known to all, compared to the proportional control, the greatest advantage of integral control is that there is no need for a very large proportional *K*_Vh_ to realize zero static error control. If the system is stable, the controller can ideally track the dc reference signal within a finite time.

**Fig 4:**
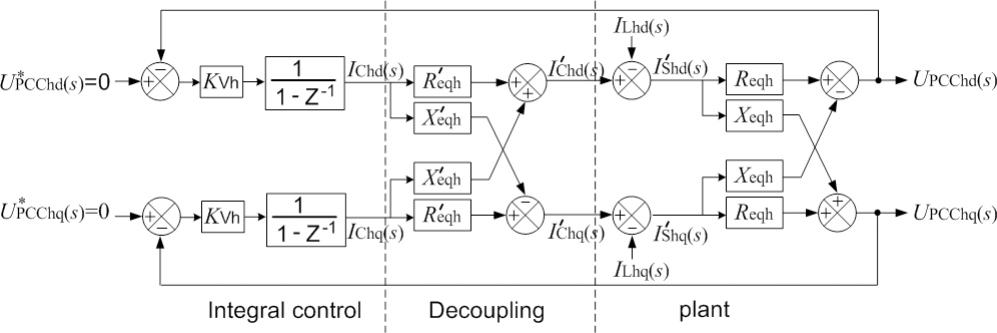
Integral-decoupling control in synchronously rotating reference frame.

Thus, the integral controller is implemented in the synchronous rotating reference frame at harmonic frequency. As *U*_PCChd_(*s*) and *U*_PCChq_(*s*) are coupled together at the synchronous rotating reference frame, this paper adds an additional decoupling control loop following the integral controller to improve the controller’s performance, as shown in Fig 4. Wherein,

*R*_eqh_ is the parallel equivalent resistance, real part of *Z*_eqh_;

*X*_eqh_ is the parallel equivalent reactance, imaginary part of *Z*_eqh_;

$\frac{1}{1-Z^{-1}}$ is the transfer function for the discrete integral controller.

### D.Parameters design for impoved R-APF algorithm

The closed-loop transfer function of the system in Fig 4 is

$$U_{PCChd}(s)=U_{PCChd}^{*}(s)\frac{A^{2}+B^{2}-A}{A^{2}+B^{2}}-U_{PCChq}^{*}(s)\frac{B}{A^{2}+B^{2}}+I_{Lhd}(s)\frac{X_{eqh}∙B-R_{eqh}∙A}{A^{2}+B^{2}}+I_{Lhq}(s)\frac{R_{eqh}∙B-X_{eqh}∙A}{A^{2}+B^{2}},$$ (9)

$$U_{PCChq}(s)=U_{PCChq}^{*}(s)\frac{A^{2}-B^{2}-A}{A^{2}+B^{2}}-U_{PCChd}^{*}(s)\frac{B}{A^{2}+B^{2}}-I_{Lhd}(s)\frac{X_{eqh}∙A+R_{eqh}∙B}{A^{2}+B^{2}}-I_{Lhq}(s)\frac{R_{eqh}∙A-X_{eqh}∙B}{A^{2}+B^{2}}.$$ (10)

Where,

$$A=1+\frac{K_{Vh}}{1-Z^{-1}}∙\left(R_{eqh}∙R_{eqh}^{\prime }+X_{eqh}∙X_{eqh}^{\prime }\right),$$

$$B=\frac{K_{Vh}}{1-Z^{-1}}∙(R_{eqh}∙X_{eqh}^{\prime }-X_{eqh}∙R_{eqh}^{\prime }).$$

The characteristic polynomial equation is

$$A^{2}+B^{2}=0.$$ (11)

Solved Eq (11) gets the closed-loop poles of the system,

$$Z=1-K_{Vh}∙\left[(R_{eqh}∙R_{eqh}^{\prime }+X_{eqh}∙X_{eqh}^{\prime })\pm j∙(R_{eqh}∙X_{eqh}^{\prime }-X_{eqh}∙R_{eqh}^{\prime })\right].$$ (12)

Assume

$$K_{Vh}^{\prime }=K_{Vh}∙\sqrt{R_{eqh}^{2}+X_{eqh}^{2}}∙\sqrt{R_{eqh}^{\prime 2}+X_{eqh}^{\prime 2}},\;\sin\left(\theta_{1}\right)=\frac{R_{eqh}^{\prime }}{\sqrt{R_{eqh}^{\prime 2}+X_{eqh}^{\prime 2}}},\;\sin\left(\theta_{2}\right)=\frac{R_{eqh}^{\prime }}{\sqrt{R_{eqh}^{2}+X_{eqh}^{2}}},$$

Eq (12) can be written as,

$$Z=1-K_{Vh}^{\prime }∙e^{\pm j(\theta_{1}-\theta_{2})}.$$ (13)

**Fig 5:**
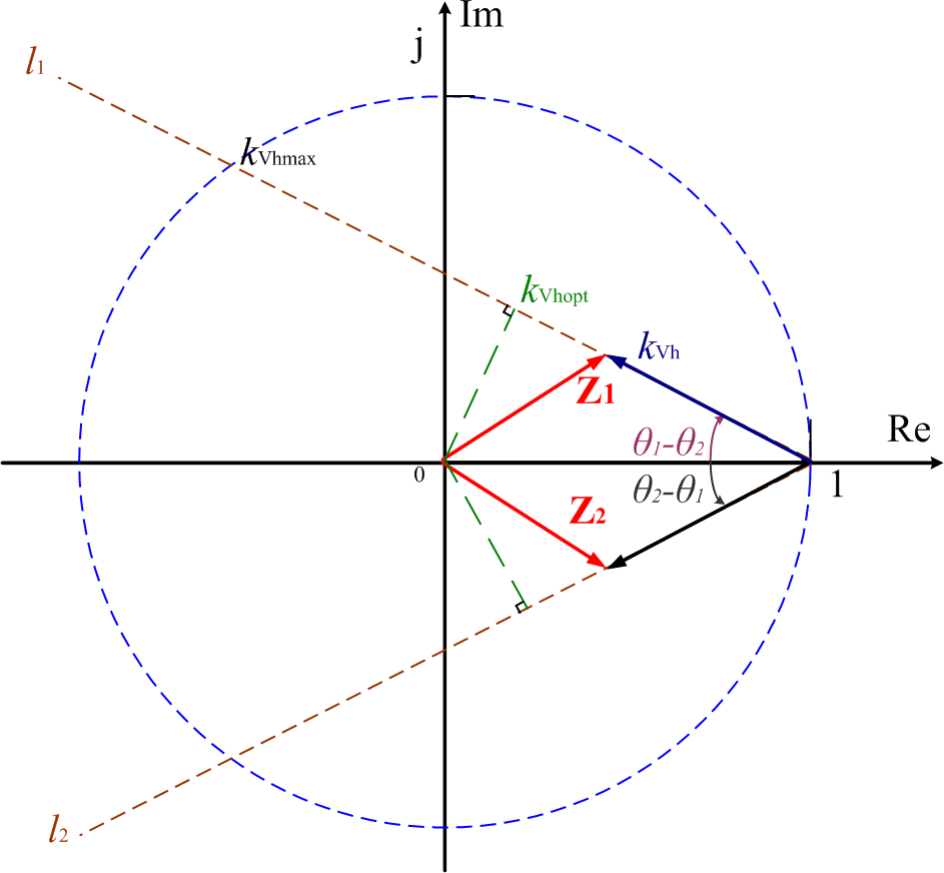
The poles of close-loop transfer function.

According to Eq (13), the close-loop poles’ trajectory can be shown as Fig 5. When *K'*_*Vh*_ is zero, the system has a pair of identical closed-loop poles at point of +1. If *K'*_*Vh*_ increases from zero, the closed-loop poles will move symmetrically towards left along the straight lines *l*_1_ and *l*_2_. And, the angle between the straight lines and the negative direction of real-axis is *θ*_1_−*θ*_2_. Without decoupling control, this angle is determined by the equivalent impedance *Z*_eqh_. With completely decoupled control, the angle becomes zero.

As is known to all, to ensure the discrete system’s stability, all the poles at the Z plane should be placed within the unit circle. And the smaller the modulus of the closed-loop pole is, the faster state variables in the system converge. Based on these stability analysis guidelines, the added decoupling control can extend the system’s stability range and accelerate the convergence. E.g. If *Z*_eqh_ is purely inductive and decoupling control is not added, the close-loop poles’ trajectory will be perpendicular to the real axis at the point of +1. In this case, no matter what value *K'*_*Vh*_ takes, the system is always unstable. But if decoupled control is adopted, whatever the value of *Z*_*eqh*_ is, the system can always be stable and converge quickly as long as *K'*_*Vh*_ is set to approximately 1.

However, the equivalent impedance of loads and systems fluctuate all the time and their exact values are not available. Thus, only partial decoupling control with an estimated value of *Z'eqh* can be used. With proper estimated parameters, the angle difference *θ*_1_−*θ*_2_ would be relatively small. And when *K'*_*Vh*_ increases from 0, the closed-loop poles will move symmetrically towards the left side along the straight line *l*_1_ or *l*_2_ and their moduli continuously decrease. The system becomes stable and its convergence speed will also continuously increase until *K'*_*Vh*_ reaches the optimal value *K*′_Vhopt_ = cos(*θ*_1_−*θ*_2_), which is slightly smaller than 1. When *K'*_*Vh*_ is set to *K'*_Vhopt_, the closed-loop poles’ moduli take the minimum value and the system achieves the optimal control performance. After that, if *K'*_*Vh*_ keeps increase, the closed-loop poles’ moduli will increase rather than go down and the control performance begins deteriorating. Once *K'*_*Vh*_ is equal or greater than *K*′_Vhmax_ = 2cos(*θ*_1_−*θ*_2_), the closed-loop poles will be out of the unit circle and the system becomes unstable again. In conclusion, it is still practical to set *K'*_*Vh*_ to 1 as it is very close to the optimal value *K'*_*Vhopt*_, and the system can be stable with a satisfying convergence speed.

## Computer Studies

### A.Comparison of R-APF and Improved R-APF Algorithm

To simplify the comparison process, the circuit displayed in Fig 3A) is used as the simulation model. Assume all the impedance *Z*_Sh_ and *Z*_Lh_ are pure resistance, and the circuit has only *h*^th^ harmonic frequency components. In this case, there is no need to do harmonic detection and decoupling control, Eqs (2) and (8) can directly applied to the instantaneous value of *i*_C_ and *u*_PCC_.

**Fig 6:**
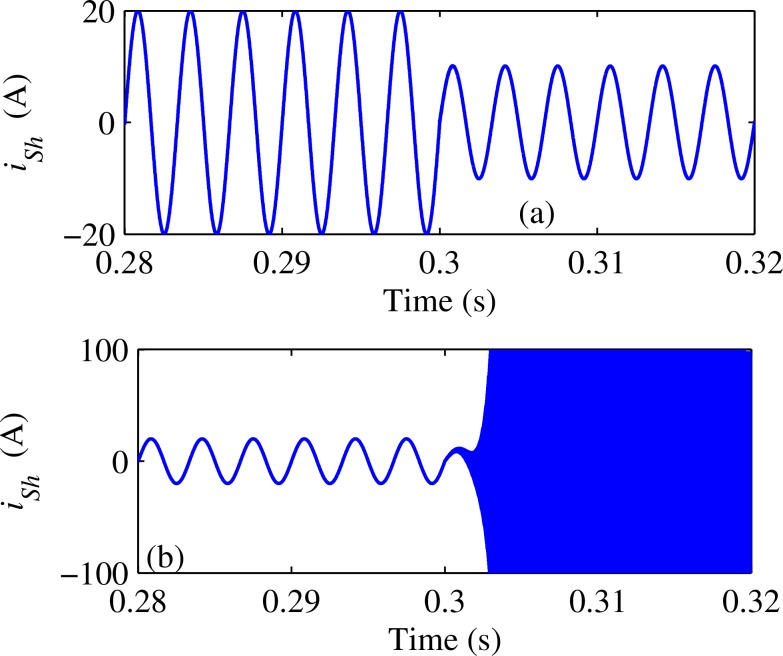
Performance of traditional R-APF algorithm: (a) With *K*_Vh_ set to 0.95/*Z*_eqh_; (b) With *K*_Vh_ set to 1.05/*Z*_eqh_.

**Fig 7:**
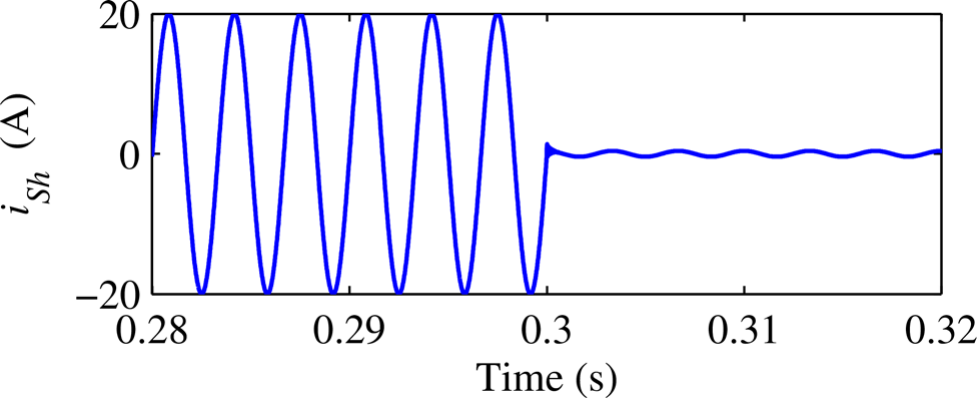
Performance of Improved R-APF algorithm With *K*_Vh_ set to 0.95/*Z*_eqh_.

Fig 6 shows the instantaneous value of system harmonic current *i*_Sh_ before and after compensated by the traditional R-APF algorithm. It is obvious that traditional R-APF can only filter out half of the load harmonic currents at most, as shown in Fig 6(A). If *K*_Vh_×*Z*_eqh_ keeps increasing and becomes larger than 1, the system harmonic current will diverge instead of decrease as shown in Fig 6(B). On the contrary, the improved R-APF can filter out almost all the load harmonic currents with a relative small *K*_Vh_, as shown in Fig 7. In this paper, the characteristic harmonic of nonlinear load, i.e. h^th^, is set to 5^th^, *Z*_Sh_ and *Z*_Lh_ are set to 1.2Ω and 6Ω respectively. But the conclusion is not confined to these particular set of simulation parameters, instead it can be extended to any other general parameters.

### B.Verify the effect of Decoupling Link

**Fig 8:**
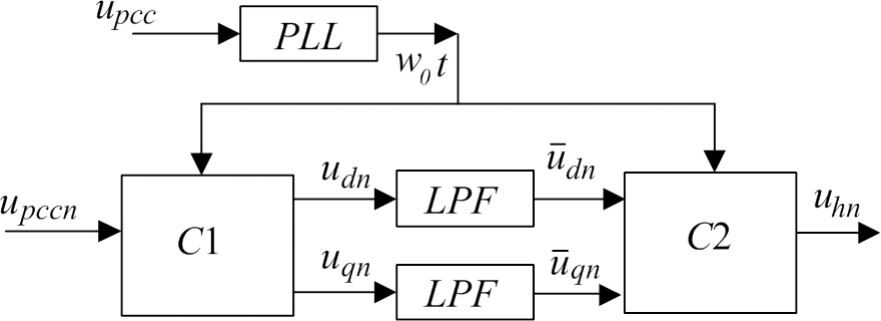
Sing-order Single-phase harmonic detection algorithm.

**Fig 9:**
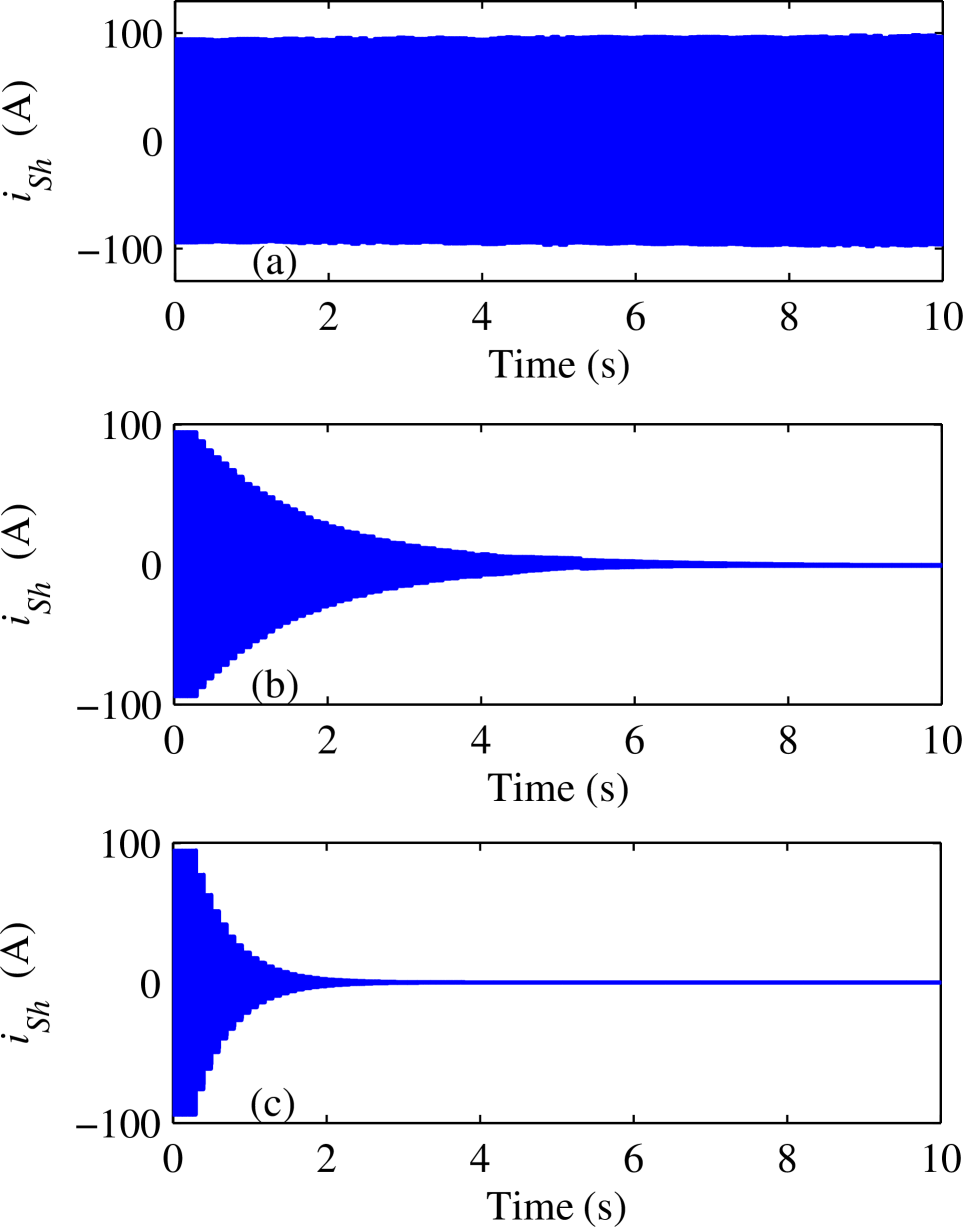
Purely inductive circuit compensate by improved R-APF: (a) Without decoupling by set θ_1_ to 0°; (b) With partial decoupling by set θ_1_ to 45°; (c) With completely decoupling by set θ_1_ to 90°.

In order to verify the effect of decoupling link, let’s assume all the impedance *Z*_Sh_ and *Z*_Lh_ are purely inductive. Here Z_sh_ and Z_Lh_ are set to j1.2Ω and j6Ω respectively, *K*_Vh_ is set to 0.95, and single-phase single-order harmonic detection algorithm (as shown in Fig 8 [14][15]) is adopted. Fig 9 shows improved R-APF’s performance under this extreme test condition. It clearly shows that without decoupling loop (*R*'_eqh_ and *X*'_eqh_ are set to 1Ω and0Ωrespectively), the load harmonic currents cannot be filtered out at all. While by partial decoupling control (*R*'_eqh_ and *X*'_eqh_ are both set to 0.707Ω), system harmonic currents are reduced to nearly zero within less than 8 seconds. And finally with completely decoupling (*R*'_eqh_ and *X*'_eqh_ are set to 0Ω and 1Ωrespectively, the exactly true value of *Z*_eqh_), the system harmonic currents are reduced to almost zero in less than 4 seconds, the fastest converge speed among the three algorithms. These simulation results confirmed that decoupling control can effectively improve the controller’s convergence speed and extend its stability region.

Where,

$${\text{C}}_{1}=\left[\begin{array}{c} \cos(h\omega_{0}t)\\ \sin(h\omega_{0}t) \end{array}\right];$$

$${\text{C}}_{2}=\left[\begin{array}{cc} 2\cos(h\omega_{0}t) & -2\sin(h\omega_{0}t) \end{array}\right];$$

n is a, b, c respectively;

h is the specific harmonic order for detection;

*ω*_0_ is the fundamental frequency of the ac system.

### C.Compensation effects for the example building

**Fig 10:**
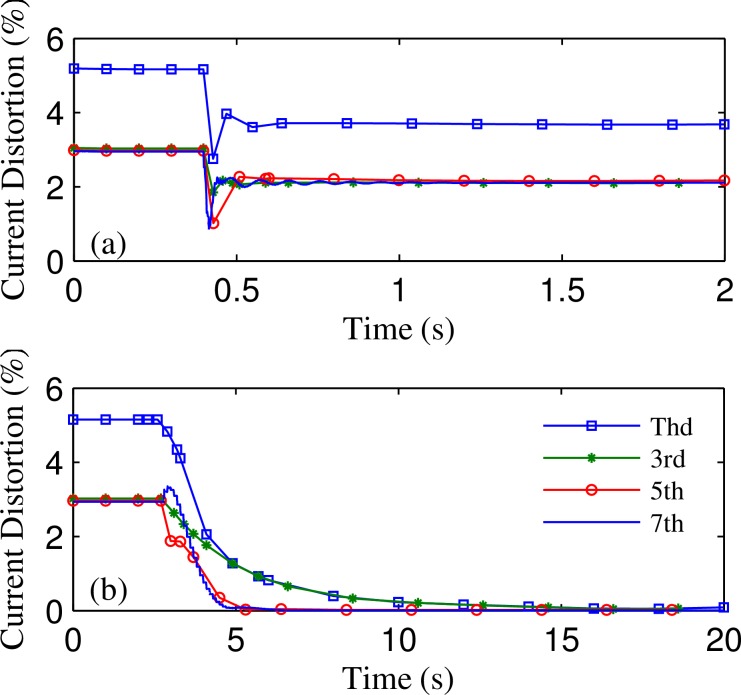
System current distortion dynamics during the compensation progress: (a) Compensated by traditional R-APF; (b) Compensated by improved R-APF.

Finally, we applied both traditional and improved R-APF algorithm to the simulation model shown in Fig 2. The dominant harmonics of non-linear load and non-linear load II are both set to third, fifth, and seventh orders, and the three dominant harmonic current values are all set equal to 10A according to the average weekday’s recorded data from the smart meter [16]. As the system harmonic voltage is very small compared with the fundamental voltage (The total harmonic distortion of U_PCC_ is less than 1%), Butterworth low pass filter (with cut off frequency at 5 Hz) is connected in series to the slide cycle average filter to realize the LPF link for accurate harmonic detection. The simulation results are shown in Fig 10. It shows that improved R-APF can filter out much more non-linear load’s harmonic current, but converges slower than the traditional R-APF due to the integral process. However, the required convergence time is less than 15 seconds which is still within the acceptable range for most harmonic elimination applications.

## Conclusion

This paper presented an investigation into the active harmonic elimination algorithm which is based on voltage detection and suitable for DG interfacing converters. It is found out that although traditional proportional R-APF algorithm can extend to eliminate harmonic current injected by non-linear loads, its performance is constrained by stability problems and can at most eliminate half of the load harmonic currents. Thus, inspired by the iterative HVC algorithm, this paper proposed a novel improved R-APF algorithm based on integral-decoupling control in the synchronous rotating reference frame at each harmonic frequency. By theoretically analyzing the trajectory of the discrete system’s closed-loop poles, the benefits of decoupling control and the design guidelines for integral coefficient are both clearly demonstrated. Simulation results confirm that the proposed harmonic elimination algorithm can effectively mitigate all the load harmonic currents within 15 seconds.
